# The help-seeking experiences of adolescents and youth with endometriosis: a systematic review and thematic synthesis

**DOI:** 10.1186/s12905-026-04566-0

**Published:** 2026-05-26

**Authors:** Sophie Meagher, Jacqueline Frayne, Jodi Renshaw-Todd, Jeneva Ohan

**Affiliations:** 1 School of Psychological Science, University of Western Australia, Perth, Western Australia; 2 Medical School, University of Western Australia, Perth, Western Australia

**Keywords:** Endometriosis, Help-seeking behaviour, Adolescent, Stigma, Health behaviours, Physician-patient relations, Patient perspectives, Qualitative research

## Abstract

**Background:**

Obtaining a diagnosis of endometriosis is often a long and involved journey. Little specific information exists about this journey for young people. This qualitative systematic review and thematic synthesis aimed to describe help-seeking experiences for young people who have been diagnosed with endometriosis, with attention to their needs, priorities, and relationships.

**Methods:**

A systematic search was conducted of MEDLINE, Embase, PsycINFO, CINAHL, SocINDEX, Google Scholar, and ProQuest Dissertations and Theses on 18/12/2024. This combined the term “endometriosis” with variants of the concept “help-seeking”. Eligible articles reported the first-person perspectives of young people aged 10–24 years who were diagnosed with endometriosis. Quality assessment was conducted using the QualSyst tool. Thematic synthesis was used to construct themes.

**Results:**

A total of 5,287 unique titles were assessed. 29 qualitative or mixed-methods studies (reported in 35 articles) met inclusion. Five analytical themes were generated: (1) Distinguishing ‘normal’ from ‘abnormal’ menstruation is difficult for young people, (2) Young people anticipate unhelpful responses, (3) Healthcare encounters aren’t tailored to young people, (4) Invalidation has disruptive consequences, and (5) Young people need acknowledgment and resources.

**Conclusions:**

Validating relationships and age-relevant information and resources are needed to improve the help-seeking experiences of young people with endometriosis. Healthcare professionals must also ensure services are tailored to young people’s life stage and concerns.

**Trial registration:**

Prospero ID CRD42024575314.

**Supplementary Information:**

The online version contains supplementary material available at 10.1186/s12905-026-04566-0.

## Background

Endometriosis is a chronic inflammatory gynaecological condition characterised by tissue resembling the uterine lining growing at various locations externally to the uterus, affecting approximately one in ten women and people assigned female at birth internationally [1–3]. For two thirds of diagnosed individuals, symptom onset occurs during adolescence [4]. Symptoms have wide heterogeneity, but can include severe menstrual symptoms, chronic pelvic or abdominal pain, sexual difficulties, and sub- or infertility, which are frequently concealed by sufferers due to societal stigma [5]. Symptoms can be profoundly disruptive, and for adolescents (defined as 10–19 years of age) and youth (up to 24 years of age) [6], can impact developmentally important experiences including educational attendance [7]. Consequently, young people with endometriosis may have reduced quality of life compared to same-age peers and even older individuals with endometriosis [8, 9]. Hence, the experiences of this age demographic are unique, and this review focuses on individuals aged 10–24 years (herein termed *young people*).

Diagnostic delays of around 7 years (average) add to the burdensomeness of endometriosis [10, 11]. Diagnostic delays arise partially from medical factors including symptom overlap with other conditions and, often, the need for laparoscopic surgery to confirm a diagnosis [12]. Other barriers to timely diagnosis include social and behavioural factors such as help-seeking delays, which for young people can be up to 5 years from symptom onset to first medical consultation [4, 13]. *Help-seeking* can be defined as “problem-focused, planned behaviour, involving interpersonal interaction” [14] (p. 280). Antecedents to help-seeking include (1) recognising the problem, (2) deciding to seek help, and (3) selecting a help source, which may be formal (e.g., a healthcare professional) or informal (e.g., a parent or friend who might offer support or advice). Help-seeking is therefore a complex phenomenon with internal (e.g., decision-making) and interpersonal (e.g., communication) elements. For young people, help-seeking for health problems can be influenced by knowledge and attitudes within family, peer, educational, and healthcare systems (e.g., concepts of what is ‘normal’ or perceptions of symptoms as ‘real’ or ‘fake’) [15]. Adult support may also be needed to help young people access and navigate healthcare, particularly in adolescence [15–17]. However, these elements of young people’s help-seeking are often not clearly addressed in research.

Compared to adults with endometriosis, young people’s help-seeking experiences may be particularly prolonged and challenging. A systematic review of factors associated with diagnostic delay found that younger age at endometriosis symptom onset and younger age at first medical encounter were significantly associated with longer diagnostic delays [10]. This may arise from both delayed help-seeking and adverse help-seeking experiences, with cross-sectional evidence suggesting that individuals with adolescent-onset symptoms may wait up to three times longer to seek medical help than adults [4, 18], and are more likely to report experiences of medical dismissal (i.e., being told nothing is wrong), compared to those with adult-onset symptoms [4]. Additionally, a survey-based study indicated that younger people’s healthcare experiences may be subjectively worse than older people’s, including poorer perceptions of access to care, poorer ratings of providers’ skills, and lower ratings of overall patient-centred care [19]. Interestingly, this study did not replicate findings of longer help-seeking delays or overall diagnosis times for younger people, yet their perceptions of care were still worse overall, suggesting a need to better understand the subjective aspects of help-seeking for this demographic.

A meta-synthesis of qualitative evidence is an appropriate avenue for understanding young people’s help-seeking experiences due to its ability to highlight what is personally important [20, 21]. Existing qualitative reviews have described the phenomenon of living with endometriosis more broadly and have identified challenging healthcare encounters as a prominent theme, stemming from patients’ perceptions that symptoms were minimised as ‘women’s issues’ or psychologised and presumed to be ‘in their head’ [12, 22]. However, there has been limited attention to the experiences of specific age groups amongst these studies, resulting in an incomplete picture of endometriosis experiences for various points during development [23].

Research on young people with endometriosis is emerging. Existing reviews focusing on this age group have tended to identify factors in diagnostic delay for young people, bringing needed attention to systemic barriers such as insufficient health education [24, 25]. However, previous reviews have been either non-systematic or have not platformed young people’s own voices due to limited inclusion of primary qualitative evidence. Young people’s subjective experiences of help-seeking are crucial as these may impact not only diagnosis times, but young people’s patient-provider relationships and trust in healthcare [19]. There has also been limited attention to how other informal help sources may begin to shape young people’s help-seeking even before they present to medical services. An enhanced understanding of young people’s help-seeking could guide the development of age-appropriate resources and services, empirically grounded in young people’s needs.

The aim of this study was therefore to synthesise existing qualitative evidence on the help-seeking experiences of young people with endometriosis, with attention to their needs, priorities, and relationships. To do this, we conducted a meta-synthesis answering the question: What are the help-seeking experiences and needs of individuals aged 10–24 years old who have been diagnosed with endometriosis, from the point of symptom onset to diagnosis?

## Methods

This review used Thomas and Harden’s thematic synthesis [26], an approach to qualitative meta-synthesis based on thematic analysis [27]. This allowed findings to go beyond those of the primary studies (most of which did not focus solely on young people) and generate insights specific to this cohort, while retaining transparency by tracking the development of themes using an established method. Findings were reported using Preferred Reporting Items for Systematic Reviews and Meta Analyses (PRISMA) guidelines [28], with additional guidance from Braun and Clarke’s Reflexive Thematic Analysis Reporting Guidelines [29]. A critical realist epistemology was adopted, which assumes data are informative about a real phenomenon (e.g., help-seeking) but that these data require active interpretation to generate understandings of the psychosocial structures that give rise to them [30]. This allowed our interpretations to be contextualised by young people’s life-stage and relationships.

### Search strategy

**Fig. 1:**
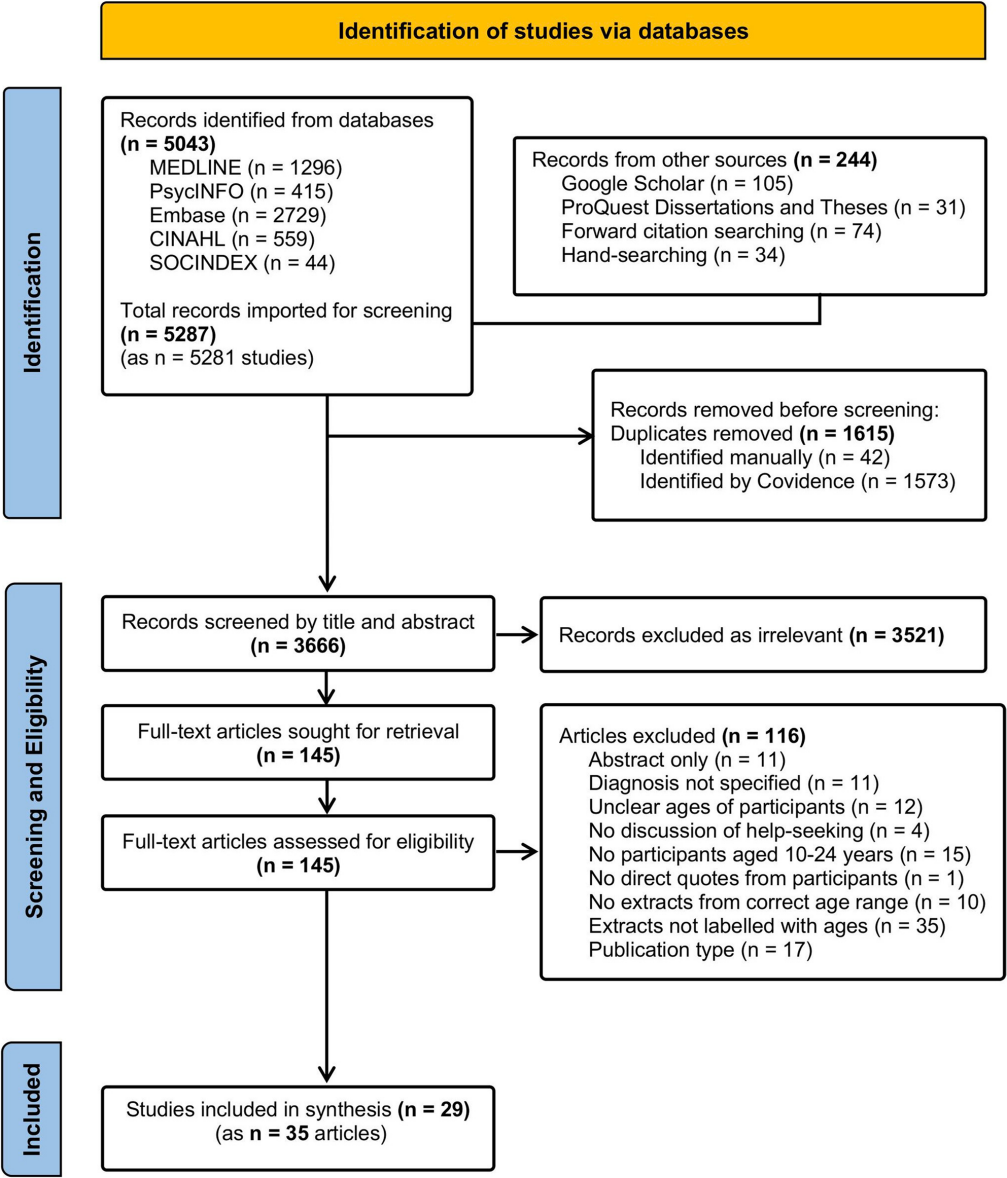
The systematic search process and final inclusion of articles

A systematic electronic search (Fig. 1) was conducted on 18/12/2024 of the databases MEDLINE, PsycINFO, and Embase using the Ovid platform, and CINAHL and SocINDEX using the Ebsco platform. A search for unpublished postgraduate-level theses, which can be rich sources of qualitative data [20], was also conducted using Google Scholar and ProQuest Dissertations and Theses. The reference lists of 14 published review articles on experiences of endometriosis were then hand-searched, and forward citation searching was performed using the “Cited by” function in Google Scholar.

The full search strategy and terms used are available in the Supplementary Materials (Appendix 1). The PICo framework (population, phenomenon of interest, and context) informed the strategy, which combined the term “endometriosis” (the context) with variations of “help-seeking” (the phenomenon). “Young people” (the population of interest) was not included as a search term to avoid prematurely excluding studies that did not focus on young people but included participants whose data could be separated out. Ages were therefore identified during screening. Numerous wordings were chosen for “help-seeking” to capture its complexity (e.g., encompassing help-seeking decisions and healthcare encounters).

The MEDLINE strategy was constructed first and validated by testing its ability to return known eligible articles. A university librarian was consulted twice, and the Peer Review of Electronic Search Strategies (PRESS) Evidence-Based Checklist was used to enhance rigour [31]. The search strategy was then adapted for each database. For the simpler interfaces of Google Scholar and ProQuest, the approach of using one complex search strategy was substituted for simplified, successive search strategies, each using a combination of terms from the original MEDLINE strategy (e.g., endometriosis AND help-seeking) and exporting the first ten pages of results each time [32].

### Eligibility criteria

Articles were included if they (1) contained primary qualitative research or mixed-methods research from which qualitative data could be extracted, (2) were available in English, and (3) included at least one participant who: (i) self-reported a medically confirmed diagnosis of endometriosis, (ii) was aged 10–24 years, and (iii) had provided a first-person account of help-seeking evidenced by a clearly attributed data extract (e.g., quotes were labelled with age and diagnostic status, and/or were attributed to a ‘young’ person with endometriosis). Studies including additional diagnoses or focusing on broader symptoms (e.g., dysmenorrhea) were excluded unless data specific to endometriosis could be separated out. Reviews were excluded but their reference lists were consulted as sources of potentially eligible articles.

### Study selection

Search results were imported to the online systematic review platform Covidence [33]. Most duplicates were removed automatically, and others manually. Two reviewers (SM and JRT, PhD students in clinical psychology and nursing, respectively) independently screened all titles and abstracts to eliminate irrelevant studies. Cornally and McCarthy’s conceptual definition of help-seeking [14] was used to determine the relevance of participants’ accounts, rather than relying on instances of the term “help-seeking”. Both then read all remaining texts in full and independently recorded the reasoning for their selection decisions, before checking agreement and resolving discrepancies collaboratively. Where indecision remained for a small number of articles, senior researchers JO (a clinical psychologist), and JF (a general practitioner) were consulted. This excluded several more articles (e.g., where diagnostic status was unclear) [34, 35].

### Critical appraisal and data extraction

SM appraised the studies using the QualSyst tool, assigning scores of 0 (no) to 2 (yes) for ten criteria, then converting the totals to study-level quality scores between 0 and 1, with 0.55 considered minimally acceptable [36]. Inter-rater agreement [36] was checked by comparing scores with those produced by a fourth-year psychology student providing temporary research assistance, returning no discrepancies. Texts were then uploaded in full to NVivo 14 qualitative analysis software [37], before data were extracted and checked for completeness and relevance by JO and JF. In Phase 1, extracted data included study characteristics (e.g., author(s) and aims). In Phase 2, extracted data included (i) young people’s verbatim quotes that had been reported in each study, and (ii) authors’ analytic statements for these quotes, typically presented alongside each quote in the results. Broader analytic findings (e.g., related to the wider study sample) were not extracted.

### Data synthesis

Thematic synthesis was led by SM in collaboration with JO and JF. SM is a provisional psychologist, PhD student in clinical psychology, and former schoolteacher. JO is a clinical psychologist and researcher with expertise in parenting, stigma, and help-seeking. JF is a general practitioner, senior lecturer, and medical researcher with expertise in women’s health. Additionally, SM and JO have lived experiences of endometriosis. Combined with their clinical backgrounds, these gave rise to simultaneous ‘insider’ and ‘outsider’ perspectives [38, 39] that they actively interrogated through reflexive journalling and collaborative refinement of themes within the multidisciplinary research team [29, 40]. Lived experience perspectives, when combined with reflexive openness, can be a resource to data synthesis, potentially increasing the relevance of findings to communities [39].

Using Thomas and Harden’s three-stage approach, SM completed inductive line-by-line coding of all data extracts, forming initial impressions of content (e.g. *‘anger and frustration*’) and meaning (e.g., ‘*ill intent sometimes ascribed to doctors’*). Codes were then grouped together to generate descriptive themes, initially forming content-based ‘topics’ (refined collaboratively with JO and JF). Per Thomas and Harden [26], these were partially informed by the research question and by conceptual knowledge about help-seeking [14], and partially data-driven, arising from distinctiveness or similarity between codes. At this stage, contrasting evidence relating to a topic (e.g., barriers and facilitators to ‘*Recognising the problem’*) formed descriptive subthemes.

Finally, SM constructed candidate ‘analytical themes’ (refined collaboratively) by forming assertions about the descriptive themes to directly address the review question [26]. At this stage, themes became interpretive ideas, rather than topics [38], hence contrasting evidence related to each descriptive theme was re-allocated to relevant analytical themes to form an interpretive narrative (e.g., barriers to *‘Recognising the problem’* became their own analytical theme *‘Distinguishing ‘normal’ from ‘abnormal’ menstruation is difficult […]’*, while facilitators were allocated to the analytical theme *‘Young people need acknowledgment and resources*). This process was aided by visual maps.

## Results

### Characteristics of included studies

Screening of 5,287 unique titles resulted in a final sample of 35 articles, reporting on 29 studies (see Table 1). Only one title referred to ‘help-seeking’ [41], but all articles contained quotes conceptually relevant to help-seeking, exploring topics such as healthcare experiences and disclosure decisions. The majority were published since 2023 (*n* = 17), with a range from 2003 to 2024. Most were conducted in Australia (*n* = 7), the United States (*n* = 5), New Zealand (*n* = 5), and the United Kingdom (*n* = 5), with others conducted in Europe (*n* = 3), Africa (*n* = 1), or elsewhere (*n* = 3). The youngest participant identified was 15 years old [42]. Some studies lacked a detailed breakdown of their sample by age, but it is estimated that collectively at least 400 young people participated (see Table 1, Column 5). Of these young people, more than 100 were directly quoted at least once, totalling approximately 260 quotes and analytic statements extracted for analysis. Most studies reported all-female samples, but in some, gender identities were unspecified (e.g., referring to ‘patients’). Three studies focused on LGBTQ+ participants [43–45], and three others reported some gender diversity.

**Table 1:** Characteristics of the 29 primary studies (35 reports) included in the systematic review

Author(s), Year of Publication, Country	Aim(s) of research	Recruitment method	Broader sample characteristics	*N* participants matching review criteria (aged 10–24 with confirmed endometriosis)	Design and orientation	Data collection and analysis	Qual-Syst score
Bergen et al., 2023 [46] Kenya	Explore perspectives of Kenyan women regarding their journeys to endometriosis diagnosis and treatment	Kenyan women with endometriosis recruited via a support group	37 women aged 22–48 years	≥ 1	Qualitative design with story-writing methodology	Anonymous online text responses analysed with deductive TA	0.75
Blunt, 2023 [47] United States	Explore how Black women in the U.S. were affected by endometriosis during the COVID-19 pandemic	Black women with endometriosis in the U.S. recruited via social media flyers and snowball sampling	8 women aged 18–37 years	1	Qualitative design with a phenomenol-ogical lens	Semi-structured interviews analysed with IPA	0.95
Cole et al., 2021 [48] United Kingdom	Examine constructions of identity in endometriosis and barriers to identity reappraisal in a context where women are expected to conform to feminine roles	Women with endometriosis recruited via the webpages of endometriosis support charities in the U.K.	34 women aged 20–56 years	3	Qualitative design with a feminist and critical realist approach	Anonymous online survey responses analysed with reflexive TA using Franks’ (2002) feminist framework	0.95
Cox et al., 2003 [49] Australia	Identify the information needs of women facing laparoscopy for endometriosis	Women with endometriosis recruited via the Endometriosis Association (Victoria) and Epworth Endometriosis Centre	61 women aged 20–64 years	5	Mixed-methods design	Focus groups held with a portion of survey respondents were analysed with TA	0.75
Davenport et al., 2024 [50] Australia	Explore how individuals with endometriosis experience sexual health communication with GPs; understand barriers and facilitators	Individuals with endometriosis recruited via social media posts by Endometriosis Australia (reposted by others)	141 participants (98% women) aged 18–52 years	≥ 1	Qualitative study within a larger mixed-methods project	Online, anonymous free-text survey responses analysed with template analysis	0.95
Eder & Roomaney, 2024a [43]; 2024b [44] International	1: Report on transgender and non-binary people’s experiences of living with endometriosis	Gender diverse individuals with endometriosis recruited internationally (e.g., U.S., New Zealand, Australia, Norway, Canada, and Ireland) through social media support groups	11 participants aged 21–31 years (2 trans-masculine, 7 non-binary, 2 both trans-masculine and non-binary)	≥ 1	Qualitative design with a phenomen-ological lens	Semi-structured online interviews and diary entries analysed with hermeneutic analysis	0.85
	2: Report on the healthcare perceptions of transgender and non-binary people with endometriosis						
Ellis et al., 2022 [51]; 2023 [52] New Zealand	1: Understand experiences of endometriosis patients in New Zealand to make future research more patient-centred	Patients in New Zealand with either confirmed or suspected (i.e. “working”) diagnoses of endometriosis recruited via social media and snowballing	50 patients aged 18–48 years, genders not specified (84% had confirmed diagnoses)	17 (34%)	Mixed-methods design with a patient-centred approach	Asynchronous, online text-based discussions analysed with an iterative thematic approach	0.90
	2: Assess needs and priorities of endometriosis patients in New Zealand and barriers to care						
Ellis et al., 2024 [53] New Zealand	Investigate the experiences of Māori and Pasifika endometriosis patients in Aotearoa, New Zealand	Māori and Pasifika participants with endometriosis recruited via social media and patient organisation networks	37 participants (27 Māori, 10 Pasifika) aged 20–55 years, genders not specified	4 (11%)	Mixed-methods design	Asynchronous, online text-based discussions analysed with inductive TA	0.80
Ellis et al., 2024 [45] New Zealand	Assess the experiences of endometriosis patients identifying as LGBTQIA + in Aotearoa, New Zealand	LGBTQIA+ patients with either confirmed or suspected (i.e. “working”) diagnoses of endometriosis recruited via social media and snowballing	28 participants aged 18–36 + years. All identified as non-heterosexual, half as gender diverse	8	Mixed-methods design	Asynchronous, online text-based discussions analysed with inductive TA	0.80
Evans et al., 2022 [54]; Katz et al., 2024 [55] Australia	1: Explore treatment use and satisfaction in Australian women with endometriosis	Australian women with endometriosis recruited from the community via social media, university forums, and gym groups	1: Text responses from 507 women aged 18–49 years	≥ 1	Mixed-methods design with a critical realist approach	(For qualitative data) Text responses to open-ended questions were analysed with template TA	1.00
	2: Explore perspectives of Australian women with endometriosis and relationship with biopsychosocial factors		2: Text responses from 383 women aged 18–49 years				
Girard et al., 2023 [56] Switzerland	Understand women’s views about endometriosis and fertility and their perception of reproductive options	French-speaking women with endometriosis in Switzerland who had participated in a previous online survey	11 women aged 19–39 years	1	Qualitative design with a grounded theory, open-inquiry approach	Semi-structured interviews analysed with thematic content analysis and the constant comparative method	0.95
Gomez et al., 2019 [57] United States	Explore perspectives on family planning among young people who perceive they may have fertility problems due to conditions such as endometriosis	Young couples in Northern California recruited via community organisations, health clinics, colleges, and social media	A subsample of 12 from the broader Young Couples study (10 female aged 18–24 years)	2	The broader Young Couples Study had a mixed-methods design	Semi-structured interviews analysed with TA incorporating collaborative coding	0.90
Hearn et al., 2024 [41] United Kingdom	Identify barriers and facilitators to effective help-seeking for endometriosis in a healthcare context and inform intervention development	Individuals with endometriosis recruited via social media or via a pool who had agreed to be contacted after previous research participation	33 survey participants aged 20–48 (genders not specified); 21 diagnosed, 12 seeking diagnosis	≥ 1	Qualitative design	Open-ended survey responses analysed using TDF and COM-B models. Interviews were held but did not involve young people	0.90
Ilschner et al., 2022 [58] Australia and France	Explore the communication experiences of women with endometriosis from symptom onset to first consultation, and from first consultation to diagnosis	Women with endometriosis in Australia and France recruited via a Facebook group (“Life with Endometriosis”), or by email invitation via EndoFrance	13 Australian and 13 French women aged 18–65 years	5	Qualitative design with a life-history approach	Semi-structured interviews were analysed with TA	0.90
Jaeger et al., 2022 [59]; Gstoettner et al., 2023 [60], Austria	1: Understand the pain experiences, coping strategies, and needs of women with endometriosis in Austria	Women with endometriosis in Austria recruited via the Endometriosis Association Austria (EVA) Facebook page	10 women aged 22–51 years	2	Qualitative design	Problem-focused interviews were analysed with qualitative content analysis	0.70
	2: Same aims						
Karavadra, 2021 [61] United Kingdom	Understand delays to diagnosis through the perspectives of women with endometriosis and healthcare professionals	Women with endometriosis and healthcare professionals recruited via a gynaecology clinic and additional channels	15 women aged 22–45 years and 15 healthcare professionals (9 female)	5	Qualitative design with a constructivist epistemology	Women’s semi-structured interviews were analysed using grounded theory	1.00
Krsmanovic & Dean, 2022 [62] United States	Investigate how women with endometriosis disclose about their disorder in the workplace	United States residents with endometriosis recruited via purposeful sampling on Reddit and Facebook	119 participants aged 20–59 years (genders not specified)	≥ 1	Qualitative design with an interpretive perspective	Open-ended, anonymous online survey responses analysed using iterative TA	0.90
Loo, 2024 [63] New Zealand	Explore the healthcare experiences of young Southeast Asian women in New Zealand (NZ) and understand their resilience	Young Southeast Asian women in NZ with various diagnoses including endometriosis recruited via social media and using flyers in a Malaysian café	14 women aged 21–35 years old	1	Qualitative design with a critical realist framework	Semi-structured online interviews were analysed with reflexive TA	1.00
Moradi et al., 2014 [64] Australia	Explore the impact of endometriosis across three age groups (16–24, 25–34, and 35 + years)	Women with endometriosis in Australia recruited via an endometriosis clinic and from the wider community via GPs	35 women aged 17–53 years	13	Qualitative design	Semi-structured focus group discussions were analysed with TA	0.85
Nielsen et al., 2023 [65] Denmark	Explore lived experiences of endometriosis in adolescence and the impact of social reactions on the illness experience	Women with endometriosis recruited via collaboration with the Danish Endometriosis Patient Association and via social media (Facebook groups)	7 women aged 21–53 years	2	Qualitative design with a critical hermeneutic approach	Semi-structured online interviews were analysed with Ricoeur’s critical interpretation theory	0.90
Plotkin, 2004 [42] United States	Understand the experiences and concerns of adolescents with endometriosis	Adolescent girls with endometriosis (most from the U.S.) recruited via physician invitation, high schools, endometriosis associations, and online support groups	16 girls aged 15–19 years (15 American, 1 Canadian)	16	Qualitative design with narrative methodology	Semi-structured interviews and photographs were analysed with categorical-content analysis (a form of narrative analysis)	1.00
Randhawa, 2023 [66] United Kingdom	Explore experiences of endometriosis among adolescents/young adults and the impact on their lives and identities (Study 1 of thesis)	Adolescents/young adults in the U.K. recruited from endometriosis support groups and social media (all identified as White British)	24 female participants aged 18–24 years (mean age = 21.1 years)	24	Qualitative study with a feminist lens (within a larger thesis)	Narrative interviews were analysed using Braun and Clarke’s TA	1.00
Requadt et al., 2023 [67] International	Examine how people with endometriosis in different age groups (18–24, 25–34, and 35+) experience the diagnostic process	Individuals with endo-metriosis from 63 countries recruited via Reddit forums (convenience sampling)	2,017 participants, 98.6% female	251	Mixed-methods design	Qualitative survey questions were analysed using TA	0.80
Seear, 2009a [68]; 2009b [69] Australia	1: Examine why women with endometriosis may not comply with health advice	Australian women with endometriosis recruited via the newsletter of an endometriosis support group and via snowballing	20 women aged 24–55 years	≥ 1	Qualitative design informed by theories of menstrual etiquette and patients as experts	Semi-structured interviews were analysed using TA incorporating Miles and Huberman’s interactive model	0.90
	2: Explore women’s concealment of menstrual problems and impact on diagnostic delay						
Taffs et al., 2024 [70] Australia	Identify and explore the unmet needs of adolescents and young adults with endometriosis	Young people with endo-metriosis recruited via social media and via a Melbourne women’s hospital	131 participants (99.3% female) aged 18–25 years	107	Qualitative, cross-sectional design	Open-ended, online survey responses analysed with template analysis	1.00
Thorpe et al., 2022 [71] United States	Explore black women’s experiences of patient-provider communication about sexual pain; describe the pathway from disclosure to treatment	Cisgender black women in the southern U.S. with a reproductive or sexual pain condition, recruited from prior survey participation	25 women aged 23–44 years	1	Qualitative study with a constructivist lens (within a mixed-methods project)	Semi-structured interviews analysed using constructivist grounded theory	0.95
Wells, 2023 [72] New Zealand	Identify the self-management strategies used by New Zealanders with endometriosis	Women with endometriosis recruited online via Endometriosis New Zealand and via snowballing	8 female participants aged 24–46 years	2	Qualitative design with a narrative inquiry approach	Semi-structured interviews analysed with Braun and Clarke’s TA	0.95
Wren & Mercer, 2022 [73] U.K.	Explore young women’s experiences of the diagnostic process and the importance of support during this process	Young women with endometriosis in the U.K. recruited via endometriosis support groups on Facebook	9 young adult women aged 18–30 years	4	Qualitative design; phenom-enological approach	Semi-structured online interviews analysed with IPA	0.90
Young et al., 2016 [74]; 2020 [75], Australia	1: Explore what women with endometriosis are told about their fertility by their providers	Women with endometriosis in Victoria recruited via a women’s magazine, queer-friendly organisations, and universities (purposive)	26 women aged 20–54 years	2	Qualitative design. Report 2 has a social constructionist perspective	In-person and telephone interviews analysed with Braun and Clarke’s TA	0.90
	2: Examine how women with endometriosis navigate knowledge and power within medical encounters						

n≥1 indicates that the exact number of young people matching inclusion criteria is unclear from the study information, but at least one was identified

*Legend*: *TA* Thematic analysis, *IPA* Interpretative Phenomenological Analysis, *TDF*  Theoretical Domains Framework, *COM-B* Capacity, Opportunity, Motivation, Behaviour

### Critical appraisal scores

QualSyst scores ranged from 0.70 to 1.00. A full critical appraisal table including criterion scores is available in the Supplementary Materials (Appendix 2). Reporting of reflexivity was a common weakness. It is acknowledged, however, that publication word limits may restrict the detail researchers provide about reflexivity, even when it has been meaningfully applied.

### Thematic synthesis

**Fig. 2:**
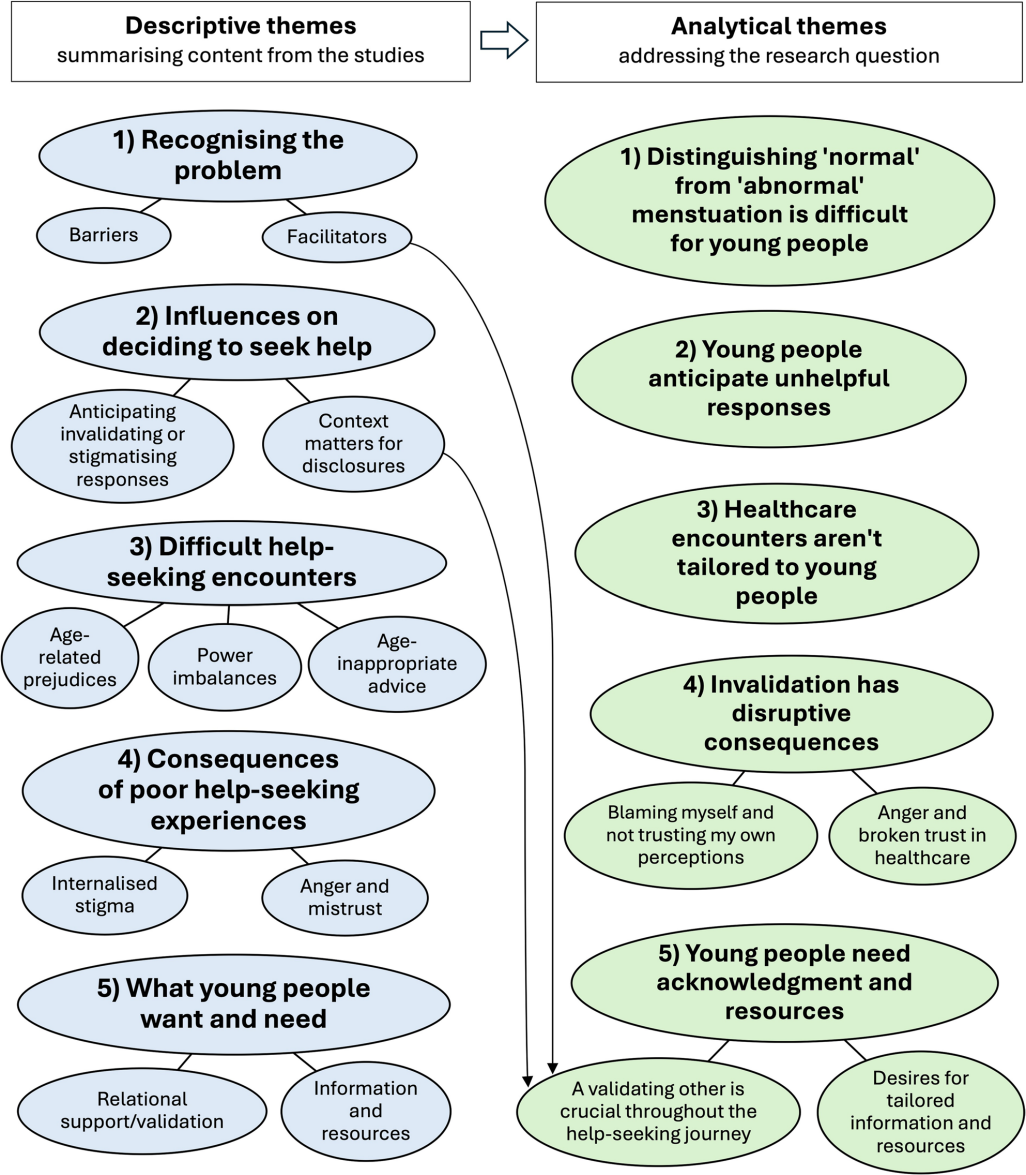
Themes generated to capture young people’s help-seeking experiences and needs

Line-by-line coding resulted in 107 initial codes. Grouping codes together generated five descriptive themes, which developed into five analytical themes, presented in Fig. 2.

Given that analytical themes extend upon descriptive themes to address the research goals [26], these are detailed below with illustrative quotes. Themes 1–2 highlight challenges initiating help-seeking, Themes 3–4 highlight medical help-seeking experiences and emotional consequences, and Theme 5 highlights needs.

### Theme 1: Distinguishing ‘normal’ from ‘abnormal’ menstruation is difficult for young people

In fifteen studies, young people described initial challenges distinguishing their symptoms from that expected of ‘normal’ menstrual pain [42, 50–52, 58, 59, 61, 63, 64, 66, 69, 70, 72, 73, 75], which delayed help-seeking. They felt they had lacked sufficient information to identify concerning symptoms: *“I was never told about to what extent the periods should hurt you*,* what extent should you let it affect your life*,* what extent do you need help?*” (age 21) [66]. Insufficient knowledge was sometimes compounded by social norms of menstrual secrecy operating within schools, making it difficult to access information to make this distinction: *“The school nurse told me to keep sanitary pads in my bag but tells you not to tell anyone. She always changed the subject when I mentioned it. Girls didn’t even talk about periods at my school”* (age 22) [61]. Although some nonetheless discussed menstruation with family or friends, these conversations were not always informative, even if sympathetic in tone; sometimes eliciting vague talk of period pain as ‘part of growing up’ or comparisons to other people’s ‘bad periods’ [42, 58, 66], positioning many to normalise their own symptoms at first.

Additionally, many recalled how they and adults close to them had lacked knowledge of endometriosis, slowing symptom identification [42, 50, 52, 64, 66, 70] (with notable exceptions, such as where mothers were diagnosed with endometriosis) [42, 72]. In some cases, myths about endometriosis (e.g., depicting it as rare or unlikely in young people) were perpetuated by adults in positions of authority or trust, such as a health teacher claiming to their class, “*I’m sure none of you have it”* (age 18–24) [52]. Without accurate information, many continued to assume their pain was normal until this perception was disrupted by a concerned other (e.g., a mother or romantic partner), a key medical event, or a worsening impact on daily life [42, 52, 58, 61, 66, 72]. For some, it became easier to recognise concerning symptoms only when they became older and engaged in self-education, or began discussing their experiences more openly: *“you listen to the news more*,* you are wiser*,* you speak up more. For me*,* abnormal was so much clearer the older I got”* (age 22) [61]. Others reported that because their symptoms worsened slowly over time, this delayed recognition, or that recognition only occurred after becoming sexually active [66].

### Theme 2: Young people anticipate unhelpful responses

In 23 studies, young people made comments suggesting that even once they recognised their own pain as severe, help-seeking was not straightforward as they often anticipated being judged or disbelieved [41–44, 47, 48, 50–55, 58, 60–63, 65, 66, 69–71, 73]. Some feared that because their pain lacked a clear ‘reason’ (pre-diagnosis), or they did not ‘look sick’ (even post-diagnosis) [42, 66], others would deem them “*incapable”* [55], *“a drama queen”* [52], or “*making excuses”* [54], particularly in school or work environments [42, 51, 55, 60–62, 66]. It felt stressful to anticipate needing to ‘prove’ their pain to peers or teachers in the context of absences [42], or to anticipate discrimination in their jobs: *“like I’m about to be fired for having a body”* (age 20) [62]. Disclosures were also impeded by fears of social rejection, particularly when symptoms interfered with activities considered typical or desirable amongst same-age peers [43, 48, 50, 61, 66]: *“I didn’t want to be like you know*,* 21 years old and have like problems having sex. That’s not what you should be*,* what society thinks you should be doing when you’re 21*” (age 21) [66]. Some worried about disclosing sexual or fertility-related concerns to romantic partners, fearing these would be seen as burdensome, particularly in new relationships [66].

Others described feeling pressure to conceal pain even from family, reporting that family members had sometimes responded unsympathetically to their pain in adolescence [42, 58, 63, 69]. Some believed this arose from relatives’ own experiences of pain dismissal:

*“when I had just absolutely severe pain […] [my mother] would be like ‘I am not going to do this every time you get your periods’ or ‘you just can’t stay in bed like this.’ And I think part of that was that she had always had very difficult periods when she was young but that there was no option*,* it was never taken that seriously”* (age 24) [69].

Distinct from interactions that modelled menstrual etiquette in public [61] or that normalised menstrual pain generally [66], such interactions were experienced as more personal, and as conveying adult expectations to “*get used to the pain; it was something I just had to live with for the rest of my life”* (age 15–19) [42]. Anticipating unhelpful responses, some actively downplayed their pain to others [42, 47, 51, 54], including parents.

### Theme 3: Healthcare encounters aren’t tailored to young people

Twenty-two studies contained young people’s accounts of difficult or invalidating healthcare encounters, ranging from explicit experiences of dismissal to receiving health advice perceived as age-inappropriate [42, 43, 45, 46, 48, 49, 51, 52, 55–58, 63–67, 69, 71, 72, 74, 75]. Many reported they were not trusted or taken seriously by health professionals, which some believed arose from prejudices about adolescents, or about young women (e.g., as attention-seeking or promiscuous):

“*[the physician] literally told [me] that I was making it up because I ‘wanted’ to have this condition so young”* (age 18–24) [67].

*“Some female doctors give you a face of judgement for having sex before marriage. They will say ‘Oh […] So you’re having sex without a partner?”* (age 22) [50].

Power imbalances were keenly felt by young people, who perceived some health professionals ‘spoke down’ to them [42], used inaccessible language [61], conveyed minimal interest in them (*“he didn’t even look at me!*”) (age 23) [61], or disregarded their fears during unfamiliar gynaecological procedures, sometimes causing them to cry [51]. They felt infantilised when they were told they were too young to have endometriosis, or were just ‘not used to’ menstrual or sexual pain [42, 49, 50, 63, 66, 67, 70, 73]. It therefore felt necessary to ‘push’ or ‘fight’ to receive help [41, 42, 44, 51, 52, 61, 64–66, 73, 75], yet some felt doctors’ attitudes rendered self-advocacy challenging: “*I feel quite personally empowered*,* but if the GP is judgmental and conservative*,* my empowerment is not enough*” (age 23) [50]. They attempted to subvert this imbalance in various ways, by changing providers, doing online research, or bringing along supportive parents as advocates [42, 61, 66, 73, 75].

Additionally, many reported health advice had felt confusing, age-inappropriate, or difficult to access [42, 44, 45, 49–52, 56, 57, 61, 63–67, 70, 73, 74]. Regarding advice to take contraceptives to manage symptoms, some disliked taking medication without knowing the cause of their pain, or felt doctors had minimised side-effects and not offered alternatives [42, 44, 51, 61, 65]. Some believed the social significance of contraception for adolescents had been overlooked: “*When I stood there as a 14-year-old and was placed on contraception*,* even though I wasn’t sexually active. I did not feel I was ready for that”* (age 23) [65]. Alternately, some reported seeking contraceptives for pain, but recalled GPs had deemed them too young and presumed they had wanted contraception for sexual activity [66]. Once diagnosed, many, including teenagers [42, 56], were advised to plan for pregnancy, which some perceived as lacking consideration of their readiness or resources: *“I’m not going to pop out a baby in the hopes of feeling better when I don′t want one […] we′re not financially stable. And what if I′m still sick after I have a baby? […] They kind of forget about that stuff”* (age 20) [74]. Conversely, others conveyed wanting fertility advice but being advised not to worry about this yet [66, 70]. Regardless of medical soundness, these experiences left young people dissatisfied with how healthcare was delivered.

### Theme 4: Invalidation has disruptive consequences

Repeated experiences of invalidation had significant consequences to young people’s self-perceptions and perceptions of healthcare, captured in 22 studies [41–43, 46–54, 61, 63, 64, 66, 67, 69, 70, 72, 73, 75].

#### Subtheme 1: Blaming myself and not trusting my own perceptions

In eight studies, young people conveyed that prior to diagnosis, they had started to internalise messages that they might be weak, ‘making it up’, or that their symptoms were psychological [41, 42, 48, 49, 51, 52, 61, 66]:

*“The doctor told my mom [.] there was no reason for me to be in pain. And I needed to see a different sort of doctor*,* a psychiatrist. I felt absolutely crazy and maybe I should see a psychiatrist”* (age 16) [42].

*“I even started to disbelieve myself […] I’d say*,* ‘Okay Mindy*,* you’re foolish. There’s nothing wrong with you*” (age 18) [42].

This was seemingly exacerbated when perceived dismissals came from multiple, or implicitly trusted adult sources: “*When you have a professional like a doctor and someone close to you like your family telling you it’s normal*,* then what am I supposed to believe?”* (age 22) [61]. This sense of having *“lost faith in”* their own perceptions (age 22) [41] felt more painful and disorienting than their initial uncertainty about whether symptoms were ‘normal’, and sometimes delayed further help-seeking [41, 42, 44]. Diagnosis alleviated these feelings, offering evidence that symptoms were not their ‘fault’ and could now be ‘proved’ [51, 66]; however, this sometimes coincided with anger.

#### Subtheme 2: Anger and broken trust in healthcare

Anger and broken trust in healthcare were conveyed in 20 studies [41, 42, 44, 47, 50–54, 61, 63, 64, 66–68, 70, 72, 73, 75]. Anger was associated with a deep sense of having been let down or deceived by those meant to offer support and guidance [51, 52], sometimes mixed with grief over an adolescence that felt ‘stolen’ [42] or ‘robbed’ [52] from them and perceptions that their suffering could have been prevented had they been taken seriously sooner. Though mainly directed at health professionals, anger sometimes extended to others:

*“[I was] tossed aside and gaslighted by many doctors*,* teachers*,* coworkers [...] when I had told someone at 16 – if I had been properly treated then*,* maybe I would not have lived through so much pain”* (age 18–24) [51].

Reflecting on the healthcare they had received pre- and post-diagnosis, many expressed negative evaluations of providers’ skills and knowledge, sometimes indicating they had needed to educate their doctors about endometriosis [42, 51, 61, 64, 66, 67]. This felt frightening as it reinforced their sense of lacking appropriate care: “*It’s difficult when adults don’t understand it. This is scary to me. Especially like doctors and nurses who are supposed to know about this sort of thing*” (age 16) [42]. Positive encounters were often described as occurring ‘finally’ after other frustrating experiences, or as fortuitous [46, 50, 51, 63, 67]: *“I was lucky enough to have a surgeon that actually knew what he was doing”* (age 18–24) [51]. This framing of good healthcare as the exception, not the rule, suggests that despite eventually receiving help, some were left with damaged trust in healthcare.

### Theme 5: Young people need acknowledgment and resources

#### Subtheme 1: A validating other is crucial throughout the help-seeking journey

In 20 studies, the crucial role of validating relationships was evident, helping young people overcome significant barriers to help-seeking [42, 44, 48, 50, 52, 54, 55, 58, 59, 61–67, 70–73]. Supportive mothers were perceived as co-interpreters and advocates who facilitated the diagnostic journey by acknowledging their pain, initiating and attending appointments, requesting referrals, and shielding them emotionally despite medical dismissals [42, 58, 66, 70, 72, 73]: *“Doctors treated me like I was insane*,* like the pain wasn’t real. I think if my mother had ever called me into question once*,* I would have just lived out the rest of my life being miserable”* (age 18) [42]. Online forums or communities of others with diagnosed or suspected endometriosis were also perceived as validating [42, 52, 61, 64, 66], helping them feel less alone and bolstering their confidence to self-advocate: *“Reading about other women’s stories online made me feel brave and strong enough to fight my doctor if I had any problems”* (age 23) [61]. However, adolescents noted these spaces sometimes lacked desired input from same-age peers, conveying that older individuals had ‘different’ problems to theirs [66]. Young people also felt empowered and validated when doctors took them seriously, acknowledged their fears, and communicated clearly and non-judgmentally [42, 44, 50, 58, 61, 66, 67]: *“he knew that I was nervous and I was afraid that he was going to cause me pain. He was very gentle and he explained everything to me”* (age 18) [42].

#### Subtheme 2:Desires for tailored information and resources

Distinct from young people’s needs for interpersonal validation were their needs for age-relevant information and resources, conveyed in 16 studies [41–45, 50–54, 61, 62, 64, 66, 70, 73]. They felt that much available information about endometriosis (including that provided post-diagnosis, or on official websites) was not targeted to them or neglected desired topics [66, 70, 73], such as how to manage their overall wellbeing: *“I thought there would be some sort of support afterwards […] on how to take care of myself and maybe things I shouldn’t be eating*,* drinking just things to manage”* (age 21) [73]. Young people therefore used social media for dual purposes, seeking not just validation of their difficulties, but expertise and recommendations based on lived experiences. Still, self-education felt burdensome at times, requiring them to sift through conflicting information [70, 73]. They also desired improvements to school health education [50, 52, 54, 64, 66, 70], and believed families and partners needed their own targeted informational resources: *“it would be good to have basic information in outsider language […] on what [endometriosis] is and what role they play and how they can help”* (age 24) [70]. Additionally, they desired tools to scaffold their own communication skills, such as by helping them make symptom-related disclosures clearly and concisely in social or work contexts [42, 66, 70]:

*“[I] wish I could comfortably say to someone ‘my chronic condition illness is flaring up. I can’t get coffee/meet you/I’ll have to reschedule’ without having to give someone a lesson about it*,* I don’t want to educate while suffering*” (age 21) [70].

## Discussion

This systematic review and meta-synthesis described the help-seeking experiences and needs of young people diagnosed with endometriosis internationally. Analytical themes conveyed experiences of struggling to make sense of initial symptoms (Theme 1), anticipating unhelpful responses (Theme 2), and feeling disempowered and invalidated within healthcare (Theme 3). For some, these experiences contributed to self-invalidation prior to diagnosis, and to anger and medical mistrust post-diagnosis (Theme 4). Needs included validation from others and tailored, age-relevant information and resources (Theme 5).

Findings overlapped partially with previous meta-syntheses of endometriosis patients’ experiences [12, 22, 76, 77], and with help-seeking barriers described for young people with menstrual or sexual health difficulties [78, 79], including themes of lacking sufficient knowledge to recognise needing help initially, and of experiencing stigma or invalidation. While past meta-syntheses have primarily focused on adult patients’ perspectives and on medical encounters, this review highlighted a multilayered relational system including peers, school staff, families, and providers with whom young people co-interpreted initial symptoms. These understandings were shaped by concepts of pain as age-normative [42, 50, 58, 63, 64, 66, 70], by age-related myths about endometriosis [42, 52, 66, 67, 73], and by adult modelling of menstrual etiquette [61] or of pain concealment [42, 69]. Experiences of pressure to conceal pain aligned partially with a theory of pain-related stigma in adolescents, whereby diagnostic uncertainty and pain invisibility can elicit disbelief and judgments of incapability from others (including family), positioning adolescents to conceal pain intentionally [15, 80]. However, there were differences; our findings suggested family responses also linked to family members’ *own* experiences of either symptom dismissal [42, 69] or validation [42, 72], not just their perceptions of adolescents’ symptoms. Hence, family members’ help-seeking experiences may shape whether (or how quickly) medical help is sought for adolescent endometriosis. Other help-seeking challenges might apply in youth. Beyond pain-related and menstrual stigma, endometriosis relates to sexual and infertility stigma [50, 81]. Anticipated or experienced stigma might therefore become increasingly complex for some young people across development, requiring stage-specific disclosure resources (e.g., to communicate symptoms in adolescence, navigate sexual or fertility discussions in youth, and self-advocate in ongoing educational or work contexts) [64, 66, 70].

The themes highlighted young people’s coping with invalidation from others, which differed between relational contexts and pre- and post-diagnosis. In the context of reported medical dismissals, family relationships were important, either shielding young people emotionally [42, 66] or exacerbating self-doubts about symptoms’ legitimacy [61]. Online communities also bolstered some young people emotionally (helping them feel ‘brave’, whereas parental validation helped them feel protected) [61]. Hence, validating relationships may moderate the impacts of invalidation from others. Post-diagnosis, they often felt angry about previous experiences of invalidation, especially by doctors perceived as either ignorant or withholding information [41, 42, 44, 51, 52, 61, 66, 68, 70, 72]. Although anger is also commonly reported among adult endometriosis patients [76], there were some differences in how frustrated emotions arose, which might partially explain previous evidence of worse perceptions of care in younger patients [19]. Notably, healthcare often felt difficult to navigate or inadequately tailored to them due to their age [42, 66, 70, 73]. Frustrated emotions might additionally have arisen from feeling marginalised socially, both amongst same-age peers (e.g., due to self-perceptions of age-incongruent symptoms) and sometimes amongst others with endometriosis (e.g., due to support groups containing mostly adults) [66]. Collectively, these findings conveyed young people’s sense of *epistemic and affective injustices* [82], or perceptions that identity-related factors, including age, had repeatedly challenged their health-related interpretive power, credibility, and self-advocacy.

### Clinical and research implications

These findings indicate young people’s help-seeking experiences are shaped by relationships and co-constructed knowledge across multiple system levels. The Minority Family Stress Model (MFSM) [83], informed by ecological systems and minority stress theories [84, 85], aligns well with these findings, offering a possible framework for future testing of moderating effects or for designing multilevel and relational interventions to improve young people’s experiences. The MFSM suggests young people *and* their families experience *distal* (external) and *proximal* (internalised and intrafamilial) stressors, but these impacts are moderated by the family’s shared understandings of the young person’s (and other relatives’) difficulties, manifesting as relational patterns (e.g., level of open acknowledgment, emotional expressiveness, and collaborative decision-making). These shape families’ engagement with external supports, including healthcare. Conceptualising families not only as sources of support (or stigma) to young people [15, 85] but as adaptive units [83] can inform relational interventions aimed at empowering families to co-interpret symptoms and collaboratively seek help (e.g., young people in this review valued parents acknowledging symptoms and attending appointments) [42, 58, 66, 72]. Additionally, the MFSM positions parents’ advocacy *and* young people’s emerging self-advocacy as important targets. Though age-related differences in parental involvement were not clear from the reviewed studies, young people’s independent engagement with supports might become more important with age [61]. Tailored support groups (e.g., for adolescents, youth, parents, or families) [64] and resources incorporating lived experience narratives (e.g., codesigned by young people and families) [86] may support these outcomes.

Understanding how parents’ own experiences impact young people’s help-seeking, and how intrafamilial norms may either challenge or facilitate help-seeking, is important for aligning supports. Previous research (including studies in this review) has suggested mothers with undiagnosed menstrual symptoms might inadvertently normalise adolescent symptoms due to likening them to their own [16, 69, 87]. However, our synthesised findings and other research specifically on parents of young people with stigmatised conditions [88–90] suggest additional reasons parents might delay help-seeking besides misperceiving adolescent symptoms as ‘normal’, such as viewing help-seeking as futile [42, 69], fearing their child will be judged [88, 90], or fearing they might be judged as parents [89, 90], hence modelling concealment as a coping strategy. Regardless of protective intentions, young people may interpret family norms of symptom concealment as parental dismissal, potentially contributing to negative self-perceptions and self-silencing [15, 16, 83, 88]. Other families vulnerable to delayed help-seeking might include those with a male primary caregiver; we found little evidence of fathers’ involvement in young people’s healthcare for endometriosis. School-based programs with parent components may offer targeted means of engaging vulnerable families and connecting them to services. Some Australian and New Zealand school programs [86, 91–93] have been piloted with the aim of facilitating earlier help-seeking for symptoms suggestive of endometriosis, with some promising outcomes (e.g., student self-reports of feeling empowered, and in one study, increased presentations to a pain clinic); however, the outcomes of such programs for vulnerable families are unknown.

Finally, we suggest areas in which clinical care can be improved. Young people often experienced decision-making about contraceptive medication as confusing and uncollaborative [42, 44, 51, 61, 65], indicating they require better explanations of the role of contraceptives and should be advised of additional modalities to support their wellbeing (e.g., physiotherapy and psychology), to enable collaborative choices, meet their desires for more holistic management strategies, and reduce their burden of information-seeking [70]. Interpersonally, providers must also try to understand young people’s concerns about contraceptives, which might arise partially from age-related stigma (particularly in adolescence), or from previous invalidating healthcare experiences that challenge their ability to trust and consider health advice (i.e., *epistemic trust*) [94, 95]. Provider training on relational skills that build epistemic trust, including attending to young people’s life-stage-specific concerns, could enhance delivery of information in several areas. Fertility advice must similarly consider young people’s current priorities and resources, or they may not perceive it as credible advice [74]. Alternately, fertility advice acted upon before feeling ready may have significant consequences, hence readiness is an important factor in these discussions. Services should also consider young people’s reports of negative experiences or fears regarding gynaecological procedures (e.g., transvaginal ultrasounds) [42, 51], which can be emotionally challenging even for adults [22]. Providing developmentally appropriate emotional support, thorough informed consent, and choices (e.g., allowing a support person) are important considerations. Evidence of parents sometimes attending appointments also raises implications for ensuring age-appropriate privacy regarding sexual health disclosures [50], and for directing communication to parent-child dyads in ways that respect the emerging autonomy of the young person. Future research should aim to understand how parents’ involvement during appointments may shift or remain stable across young people’s diagnostic journeys for endometriosis, or across cultures.

### Limitations and strengths

This review has limitations. No studies contained early adolescent participants, although experiences from this life stage were recalled by older adolescents and youths. This likely reflects diagnostic delays, and some studies’ age criteria. Nonetheless, the minority of adolescent-specific studies were rich in detail, strengthening the transferability of findings to this age cohort [38]. Second, due to varied reporting of reflexivity, we had limited insight into how authors selected quotes for publication; hence, both the primary studies and our themes might potentially overrepresent negative experiences. Still, we found consistent reporting of challenging experiences internationally, indicating these are not unusual. Finally, while some studies recruited from specific cultural contexts, our ability to discuss culture was limited by the data meeting our inclusion criteria (i.e., quotes relevant to help-seeking provided by young people, and authors’ corresponding comments), which did not always highlight cultural factors. The MFSM raises considerations for how cultural marginalisation may shape intrafamilial stress and in turn, help-seeking challenges [83], underscoring the need for highly available supports to be embedded within existing systems, including schools.

## Conclusion

This meta-synthesis indicated that among young people with endometriosis, help-seeking experiences are characterised by struggling to interpret initial symptoms, anticipating invalidation or stigma from others, and navigating healthcare encounters that do not feel tailored to their life stage. Invalidating help-seeking encounters can have disruptive consequences, including self-invalidation and damaged trust in healthcare systems. Validating caregivers, online communities, and health professionals can facilitate young people’s diagnostic journeys; nonetheless, young people also need age-relevant resources to support their own self-advocacy. Findings suggest it is important for interventions and resources to target multiple layers of young people’s relational systems, and for healthcare professionals to tailor services to young people’s life stage and concerns.

## Supplementary Information

Supplementary Material 1: Appendix 1. Search strategy.

Supplementary Material 2: Appendix 2. Critical appraisal scores.

## Data Availability

The search strategy used, and a full critical appraisal table are available in the Supplementary Materials. Codes generated in NVivo during thematic synthesis are available from the corresponding author on reasonable request.
