# Supplementary material for: The help-seeking experiences of adolescents and youth with endometriosis: a systematic review and thematic synthesis
Source: BMC Womens Health. 2026 May 26;26:378. doi: 10.1186/s12905-026-04566-0 (PMC13393719; doi:10.1186/s12905-026-04566-0)
Supplement: Supplementary file 1 — Supplementary Material 1: Appendix 1. Search strategy. [file 12905_2026_4566_MOESM1_ESM.docx]

Database(s): **Ovid MEDLINE(R) ALL**1946 to December 17, 2024

Platform: Ovid

Date of search: 18/12/2024

Search Strategy:

| **#** | **Searches** | **Results** |
| --- | --- | --- |
| 1 | *Endometriosis/ or Endometriosis/di or Endometriosis/px or endometriosis.ti,ab. | 34150 |
| 2 | Help Seeking Behavior/ or Health Behavior/ or Health Care Utilization/ or Attitude to health/ or Health Care Barriers/ or Patient Satisfaction/ or Patient acceptance of health care/ or ((help or support or care or "health care" or healthcare or treatment) adj2 seek*).mp. or ((medical or clinical or care or patient or diagnostic or healthcare or "health care") adj2 (experience* or encounter* or interaction*)).mp. or ((healthcare or "health care") adj2 utili#ation).mp. or (treatment adj2 satisfaction).mp. or (patient* adj2 satisfaction).mp. | 525919 |
| 3 | Delivery of health care/ or Quality of health care/ or Physician-patient relations/ or "Attitude of health personnel"/ or Delayed diagnosis/ or (delivery adj2 (service* or care or healthcare or "health care")).mp. or ((doctor or physician or clinician or practitioner or medical professional or "health care professional" or healthcare professional or health professional or "health care provider" or healthcare provider) adj3 patient* adj3 (relation* or communicat* or interact* or encounter*)).mp. or (delay* adj2 diagnos*).mp. or diagnostic journey.mp. | 488569 |
| 4 | Disclosure/ or Interpersonal relations/ or disclos*.mp. or social network*.mp. or social experience*.mp. or communicat*.mp. | 775961 |
| 5 | 2 or 3 or 4 | 1624648 |
| 6 | 1 and 5 | 1296 |

Database(s): **APA PsycInfo**1806 to December 2024 Week 2

Platform: Ovid

Date of search: 18/12/2024
Search Strategy:

| **#** | **Searches** | **Results** |
| --- | --- | --- |
| 1 | endometriosis.af. | 2033 |
| 2 | Help Seeking Behavior/ or Health Behavior/ or Health Care Utilization/ or Health Attitudes/ or "Physical Illness (Attitudes Toward)"/ or Health Care Barriers/ or Patient Satisfaction/ or ((help or support or care or "health care" or healthcare or treatment) adj2 seek*).mp. or ((medical or clinical or care or patient or diagnostic or healthcare or "health care") adj2 (experience* or encounter* or interaction*)).mp. or ((healthcare or "health care") adj2 utili#ation).mp. or (treatment adj2 satisfaction).mp. or (patient* adj2 satisfaction).mp. | 167979 |
| 3 | Health Care Delivery/ or Quality of Care/ or Health Care Professionals/ or Health Care Services/ or Physician Patient Interaction/ or Health Personnel Attitudes/ or Medical Treatment/ or "Clinical Judgment (Medical Diagnosis)"/ or (delivery adj2 (service* or care or healthcare or "health care")).mp. or ((doctor or physician or clinician or practitioner or medical professional or "health care professional" or healthcare professional or health professional or "health care provider" or healthcare provider) adj3 patient* adj3 (relation* or communicat* or interact* or encounter*)).mp. or (delay* adj2 diagnos*).mp. or diagnostic journey*.mp. | 193014 |
| 4 | Self-Disclosure/ or Support Groups/ or Online Support Groups/ or "Interaction (Interpersonal)"/ or Interpersonal Communication/ or Caregivers/ or disclos*.mp. or social network*.mp. or social experience*.mp. or communicat*.mp. | 529523 |
| 5 | 2 or 3 or 4 | 807787 |
| 6 | 1 and 5 | 415 |

Database(s): **Embase Classic+Embase**1947 to 2024 Week 50

Platform: Ovid

Date of search: 18/12/2024
Search Strategy:

| **#** | **Searches** | **Results** |
| --- | --- | --- |
| 1 | endometriosis.ti,ab. or *Endometriosis/ or Endometriosis/di or Endometriosis/th | 48569 |
| 2 | Help seeking behavior/ or Health behavior/ or Health care utilization/ or Attitude to health/ or Patient attitude/ or Health care access/ or Patient satisfaction/ or Personal experience/ or ((help or support or care or "health care" or healthcare or treatment) adj2 seek*).mp. or ((medical or clinical or care or patient or diagnostic or healthcare or "health care") adj2 (experience* or encounter* or interaction*)).mp. or ((healthcare or "health care") adj2 utili#ation).mp. or (treatment adj2 satisfaction).mp. or (patient* adj2 satisfaction).mp. | 1011786 |
| 3 | Health care delivery/ or Health care quality/ or Person centered care/ or Doctor patient relationship/ or Physician attitude/ or Health care personnel/ or Underdiagnosis/ or Delayed diagnosis/ or (delivery adj2 (service* or care or healthcare or "health care")).mp. or ((doctor or physician or clinician or practitioner or medical professional or "health care professional" or healthcare professional or health professional or "health care provider" or healthcare provider) adj3 patient* adj3 (relation* or communicat* or interact* or encounter*)).mp. or (delay* adj2 diagnos*).mp. or diagnostic journey.mp. | 979721 |
| 4 | Support-seeking/ or Information seeking/ or Social support/ or Support group/ or Social life/ or Self help/ or Interpersonal communication/ or disclos*.mp. or social network*.mp. or social experience*.mp. or communicat*.mp. | 1236018 |
| 5 | 2 or 3 or 4 | 2843749 |
| 6 | 1 and 5 | 2729 |

Database(s): **CINAHL**

Platform: Ebsco

Date of search: 18/12/2024
Search Strategy:

| **#** | **Searches** | **Results** |
| --- | --- | --- |
| 1 | (MH Endometriosis) OR (MH Endometriosis/DI) OR (MH Endometriosis/PF) OR (TI endometriosis OR AB endometriosis) | 9528 |
| 2 | (MH Help seeking behavior) OR (MH Patient attitudes) OR (Attitude to illness) OR (MH Attitude to health) OR (MH Patient satisfaction) OR (MH Life experiences) OR ((help OR support OR care OR "health care" OR healthcare OR treatment) N2 seek*)) OR ((medical OR clinical OR care OR patient OR diagnostic OR healthcare OR "health care") N2 (experience* OR encounter* OR interaction*)) OR ((healthcare OR "health care") N2 utili?ation) OR (treatment N2 satisfaction) OR (patient* N2 satisfaction) | 377554 |
| 3 | (MH Health Care Delivery) OR (MH Patient care) OR (MH Professional-Patient Relations) OR (MH Diagnosis, delayed) OR (MH Diagnostic Errors Psychosocial Factors) OR (delivery N2 (service* OR care OR healthcare OR "health care")) OR ((doctor OR physician OR clinician OR practitioner OR "medical professional" OR "health care professional" OR "healthcare professional" OR "health professional" OR "health care provider" OR "healthcare provider") N3 patient* N3 (relation* OR communicat* OR interact* OR encounter*)) OR (delay* N2 diagnos*) OR (diagnostic journey*) | 245837 |
| 4 | (MH Communication) OR (MH Support, psychosocial) OR disclos* OR "social network*" OR "social experience*" OR communicat* | 397145 |
| 5 | 2 or 3 or 4 | 888941 |
| 6 | 1 and 5 | 559 |

Database(s): **SocINDEX**

Platform: Ebsco

Date of search: 18/12/2024
Search Strategy:

| **#** | **Searches** | **Results** |
| --- | --- | --- |
| 1 | (MH Endometriosis) OR (TI endometriosis OR AB endometriosis) | 233 |
| 2 | (MH Help-seeking behavior) OR (MH Health behavior) OR (MH Health attitudes) OR (MH Patients’ attitudes) OR (MH Health education) OR (MH Health literacy) OR (MH Patient satisfaction) OR (MH Self-efficacy) OR ((help OR support OR care OR "health care" OR healthcare OR treatment) N2 seek*)) OR ((medical OR clinical OR care OR patient OR diagnostic OR healthcare OR "health care") N2 (experience* OR encounter* OR interaction*)) OR ((healthcare OR "health care") N2 utili?ation) OR (treatment N2 satisfaction) OR (patient* N2 satisfaction) | 51155 |
| 3 | (MH Patient care) OR (MH Medical care) OR (MH Medical personnel) OR (MH Physicians) OR (MH Physician-patient relations) OR (MH Patient-professional relations) OR (MH Medical errors) OR (delivery N2 (service* OR care OR healthcare OR "health care")) OR ((doctor OR physician OR clinician OR practitioner OR "medical professional" OR "health care professional" OR "healthcare professional" OR "health professional" OR "health care provider" OR "healthcare provider") N3 patient* N3 (relation* OR communicat* OR interact* OR encounter*)) OR (delay* N2 diagnos*) OR (diagnostic journey*) | 98903 |
| 4 | (MH Interpersonal relations) OR (MH Social support) OR (MH Caregivers) OR (MH Communication) OR (MH Social stigma) OR disclos* OR "social network*" OR "social experience*" OR (TI communicat* OR AB communicat*) | 198110 |
| 5 | 2 or 3 or 4 | 316856 |
| 6 | 1 and 5 | 44 |

**Grey literature searches**

***Google Scholar (advanced search on incognito mode):***

**Date: 19/09/2024**

**Search strategy 1:** (endometriosis) AND (help-seeking OR care-seeking)
“anywhere in the article”

- 4,100 results
- First 10 pages exported for screening.

**Date: 10/10/2024**

**Search strategy 2:** (endometriosis) AND (adolescents OR young adults)
“anywhere in the article”

- 58,700 results
- First 10 pages exported for screening.

***ProQuest Dissertations and Theses Global (advanced search):***

**Date: 10/10/2024**

**Search strategy 1:** title(endometriosis) AND (help-seeking OR care-seeking)

**Source type selected:** Dissertations & Theses

- 11 results
- All results exported for screening.

**Date: 10/10/2024**

**Search strategy 2:** title(endometriosis) AND (adolescents OR young adults)
**Source type selected:** Dissertations & Theses

- 20 results
- All results exported for screening

**Backward and Forward Citation Searching**

The following review articles were used as the basis of backward and forward citation searching:

As-Sanie, S., Black, R., Giudice, L. C., Gray Valbrun, T., Gupta, J., Jones, B., Laufer, M. R., Milspaw, A. T., Missmer, S. A., Norman, A., Taylor, R. N., Wallace, K., Williams, Z., Yong, P. J., & Nebel, R. A. (2019). Assessing research gaps and unmet needs in endometriosis. *American Journal of Obstetrics and Gynecology*, *221*(2), 86–94. <https://doi.org/10.1016/j.ajog.2019.02.033>

Culley, L., Law, C., Hudson, N., Denny, E., Mitchell, H., Baumgarten, M., & Raine-Fenning, N. (2013). The social and psychological impact of endometriosis on women’s lives: A critical narrative review. *Human Reproduction Update*, *19*(6), 625–639. <https://doi.org/10.1093/humupd/dmt027>

Cunnington, S., Cunnington, A., & Hirose, A. (2024). Disregarded, devalued and lacking diversity: An exploration into women’s experiences with endometriosis. A systematic review and narrative synthesis of qualitative data. *Journal of Endometriosis and Uterine Disorders*, 100087. <https://doi.org/10.1016/j.jeud.2024.100087>

Davenport, S., Smith, D., & Green, D. J. (2023). Barriers to a timely diagnosis of Endometriosis: A qualitative systematic review. *Obstetrics & Gynecology*, *142*(3), 571–583. <https://doi.org/10.1097/AOG.0000000000005255>

De C Williams, A. C., & McGrigor, H. (2024). A thematic synthesis of qualitative studies and surveys of the psychological experience of painful endometriosis. *BMC Women’s Health*, *24*(1), 50. <https://doi.org/10.1186/s12905-023-02874-3>

Facchin, F., Buggio, L., Dridi, D., Barbara, G., & Vercellini, P. (2021). The subjective experience of dyspareunia in women with endometriosis: A systematic review with narrative synthesis of qualitative research. *International Journal of Environmental Resarch and Public Health*, *18*(22), 12112. <https://doi.org/10.3390/ijerph182212112>

Jouanny, C., Abhyankar, P., & Maxwell, M. (2024). A mixed methods systematic literature review of barriers and facilitators to help-seeking among women with stigmatised pelvic health symptoms. *BMC Women’s Health*, *24*(1), 217. <https://doi.org/10.1186/s12905-024-03063-6>

Maulenkul, T., Kuandyk, A., Makhadiyeva, D., Dautova, A., Terzic, M., Oshibayeva, A., Moldaliyev, I., Ayazbekov, A., Maimakov, T., Saruarov, Y., Foster, F., & Sarria-Santamera, A. (2024). Understanding the impact of endometriosis on women’s life: An integrative review of systematic reviews. *BMC Women’s Health*, *24*(1), 524. <https://doi.org/10.1186/s12905-024-03369-5>

Niedenfuehr, J., & King, L. M. (2024). The Barriers That Adolescents and Young Adults with Endometriosis Experience in the United States: A Conceptual Review and Model. *Sexuality Research and Social Policy*. <https://doi.org/10.1007/s13178-024-00972-x>

O’Hara, R., Rowe, H., & Fisher, J. (2019). Self-management in condition-specific health: A systematic review of the evidence among women diagnosed with endometriosis. *BMC Women’s Health*, *19*(1), 80. <https://doi.org/10.1186/s12905-019-0774-6>

Pettersson, A., & Berterö, C. M. (2020). How Women with Endometriosis Experience Health Care Encounters. *Women’s Health Reports*, *1*(1), 529–542. <https://doi.org/10.1089/whr.2020.0099>

Robstad, N., Paulsen, A., Vistad, I., Hott, A. C., Hansen Berg, K., Øgård‐Repål, A., Rabben, J., Wallevik Kristoffersen, E., & Rohde, G. (2024). Experiences of pain communication in endometriosis: A meta‐synthesis. *Acta Obstetricia et Gynecologica Scandinavica*, aogs.14995. <https://doi.org/10.1111/aogs.14995>

Simpson, C. N., Lomiguen, C. M., & Chin, J. (2021). Combating Diagnostic Delay of Endometriosis in Adolescents via Educational Awareness: A Systematic Review. *Cureus*. <https://doi.org/10.7759/cureus.15143>

Young, K., Fisher, J., & Kirkman, M. (2015). Women’s experiences of endometriosis: A systematic review and synthesis of qualitative research. *Journal of Family Planning and Reproductive Health Care*, *41*(3), 225–234. <https://doi.org/10.1136/jfprhc-2013-100853>
