# Supplementary material for: The help-seeking experiences of adolescents and youth with endometriosis: a systematic review and thematic synthesis
Source: BMC Womens Health. 2026 May 26;26:378. doi: 10.1186/s12905-026-04566-0 (PMC13393719; doi:10.1186/s12905-026-04566-0)
Supplement: Supplementary file 2 — Supplementary Material 2: Appendix 2. Critical appraisal scores. [file 12905_2026_4566_MOESM2_ESM.docx]

**Table 2.** QualSyst scores for each of the 29 studies (as 35 reports) included in the review

| **Author(s), Year** | **Country** | **1) Objective** | **2) Design** | **3) Context** | **4) Theoretical framework** | **5) Sampling strategy** | **6) Data collection** | **7) Data analysis** | **8) Verification procedures to establish credibility** | **9) Conclusions supported by results** | **10) Reflex-ivity** | **Score** |
| --- | --- | --- | --- | --- | --- | --- | --- | --- | --- | --- | --- | --- |
| Bergen et al., 2023 | Kenya | Yes (2) | Yes (2) | Yes (2) | Yes (2) | Yes (2) | Yes (2) | Partial (1) | No (0) | Yes (2) | No (0) | 0.75 |
| Blunt, 2023  (PhD thesis) | United States | Yes (2) | Yes (2) | Yes (2) | Yes (2) | Yes (2) | Yes (2) | Yes (2) | Yes (2) | Yes (2) | Partial (1) | 0.95 |
| Cole et al., 2021 | United Kingdom | Yes (2) | Yes (2) | Yes (2) | Yes (2) | Yes (2) | Yes (2) | Yes (2) | Yes (2) | Yes (2) | Partial (1) | 0.95 |
| Cox et al., 2003 | Australia | Yes (2) | Yes (2) | Yes (2) | No (0) | Yes (2) | Yes (2) | Partial (1) | Yes (2) | Partial (1) | Partial (1) | 0.75 |
| Davenport et al., 2024 | Australia | Yes (2) | Yes (2) | Yes (2) | Partial (1) | Yes (2) | Yes (2) | Yes (2) | Yes (2) | Yes (2) | Yes (2) | 0.95 |
| Eder & Roomaney,  2024a; 2024b (2 reports) | Intern-ational | Yes (2) | Yes (2) | Yes (2) | Yes (2) | Yes (2) | Yes (2) | Yes (2) | No (0) | Yes (2) | Partial (1) | 0.85 |
| Ellis et al.,  2022; 2023 (2 reports) | New Zealand | Yes (2) | Yes (2) | Yes (2) | Yes (2) | Yes (2) | Yes (2) | Yes (2) | Yes (2) | Yes (2) | No (0) | 0.90 |
| Ellis et al., 2024 (Māori and Pasifika focus) | New Zealand | Yes (2) | Yes (2) | Yes (2) | No (0) | Yes (2 | Yes (2) | Yes (2) | Yes (2) | Yes (2) | No (0) | 0.80 |
| Ellis et al., 2024 (LGBT+ focus) | New Zealand | Yes (2) | Yes (2) | Yes (2) | No (0) | Yes (2) | Yes (2) | Yes (2) | Yes (2) | Yes (2) | No (0) | 0.80 |
| Evans et al., 2022;  Katz et al., 2024  (2 reports) | Australia | Yes (2) | Yes (2) | Yes (2) | Yes (2) | Yes (2) | Yes (2) | Yes (2) | Yes (2) | Yes (2) | Yes (2) | 1.00 |
| Girard et al., 2023 | Switzer-land | Yes (2) | Yes (2) | Yes (2) | Partial (1) | Yes (2) | Yes (2) | Yes (2) | Yes (2) | Yes (2) | Yes (2) | 0.95 |
| Gomez et al., 2019 | United States | Yes (2) | Yes (2) | Yes (2) | Yes (2) | Yes (2) | Yes (2) | Yes (2) | Yes (2) | Yes (2) | No (0) | 0.90 |
| Hearn et al., 2024 | United Kingdom | Yes (2) | Yes (2) | Yes (2) | Yes (2) | Yes (2) | Yes (2) | Yes (2) | Yes (2) | Yes (2) | No (0) | 0.90 |
| Ilschner et al., 2022 | Australia and France | Yes (2) | Yes (2) | Yes (2) | Yes (2) | Yes (2) | Yes (2) | Yes (2) | Yes (2) | Yes (2) | No (0) | 0.90 |
| Jaeger et al., 2022;  Gstoettner et al., 2023 (2 reports) | Austria | Yes (2) | Yes (2) | Yes (2) | Partial (1) | Yes (2) | Yes (2) | Partial (1) | No (0) | Yes (2) | No (0) | 0.70 |
| Karavadra, 2021, (PhD thesis) | United Kingdom | Yes (2) | Yes (2) | Yes (2) | Yes (2) | Yes (2) | Yes (2) | Yes (2) | Yes (2) | Yes (2) | Yes (2) | 1.00 |
| Krsmanovic & Dean, 2022 | United States | Yes (2) | Yes (2) | Yes (2) | Yes (2) | Yes (2) | Yes (2) | Yes (2) | Yes (2) | Yes (2) | No (0) | 0.90 |
| Loo, 2024  (Masters thesis) | New Zealand | Yes (2) | Yes (2) | Yes (2) | Yes (2) | Yes (2) | Yes (2) | Yes (2) | Yes (2) | Yes (2) | Yes (2) | 1.00 |
| Moradi et al., 2014 | Australia | Yes (2) | Yes (2) | Yes (2) | Partial (1) | Yes (2) | Yes (2) | Yes (2) | Yes (2) | Yes (2) | No (0) | 0.85 |
| Nielsen et al., 2023 | Denmark | Yes (2) | Yes (2) | Yes (2) | Yes (2) | Yes (2) | Yes (2) | Yes (2) | Yes (2) | Yes (2) | No (0) | 0.90 |
| Plotkin, 2004  (PhD thesis) | United States | Yes (2) | Yes (2) | Yes (2) | Yes (2) | Yes (2) | Yes (2) | Yes (2) | Yes (2) | Yes (2) | Yes (2) | 1.00 |
| Randhawa, 2023 (PhD thesis) | United Kingdom | Yes (2) | Yes (2) | Yes (2) | Yes (2) | Yes (2) | Yes (2) | Yes (2) | Yes (2) | Yes (2) | Yes (2) | 1.00 |
| Requadt et al., 2023 | International (60% US) | Yes (2) | Yes (2) | Yes (2) | Partial (1) | Partial (1) | Yes (2) | Partial (1) | Yes (2) | Yes (2) | Partial (1) | 0.80 |
| Seear, 2009a; 2009b (2 reports) | Australia | Yes (2) | Yes (2) | Yes (2) | Yes (2) | Yes (2) | Yes (2) | Yes (2) | Yes (2) | Yes (2) | No (0) | 0.90 |
| Taffs et al., 2024 | Australia | Yes (2) | Yes (2) | Yes (2) | Yes (2) | Yes (2) | Yes (2) | Yes (2) | Yes (2) | Yes (2) | Yes (2) | 1.00 |
| Thorpe et al., 2022 | United States | Yes (2) | Yes (2) | Yes (2) | Yes (2) | Yes (2) | Yes (2) | Yes (2) | Yes (2) | Yes (2) | Partial (1) | 0.95 |
| Wells, 2023 (Masters thesis) | New Zealand | Yes (2) | Yes (2) | Yes (2) | Partial (1) | Yes (2) | Yes (2) | Yes (2) | Yes (2) | Yes (2) | Yes (2) | 0.95 |
| Wren & Mercer, 2022 | United Kingdom | Yes (2) | Yes (2) | Yes (2) | Partial (1) | Yes (2) | Yes (2) | Yes (2) | Yes (2) | Yes (2) | Partial (1) | 0.90 |
| Young et al., 2016; 2020 (2 reports) | Australia | Yes (2) | Yes (2) | Yes (2) | Yes (2) | Yes (2) | Yes (2) | Yes (2) | Yes (2) | Yes (2) | No (0) | 0.90 |
